# Environmental drivers and transcriptomic variations shaping Lei bamboo shoots across cultivation regions

**DOI:** 10.3389/fpls.2025.1565665

**Published:** 2025-04-01

**Authors:** Yingzhai Qian, Jingyi Jia, Zhenlin Chen, Kecheng Wang, Peng Li, Peijun Gao, Yeqing Ying, Wenhui Shi

**Affiliations:** ^1^ National Key Laboratory for Development and Utilization of Forest Food Resources, Zhejiang A&F University, Hangzhou, China; ^2^ Key Laboratory of Bamboo Science and Technology of Ministry of Education, Bamboo Industry Institute, Zhejiang A&F University, Hangzhou, China

**Keywords:** bamboo shoots, environmental factors, nutritional traits, transcriptome, precision cultivation, regional management

## Abstract

The nutritional composition of bamboo shoots varies significantly across regions, yet the precise environmental drivers and underlying molecular mechanisms remain poorly understood. In particular, the influence of soil properties and climatic factors on key metabolic pathways regulating bamboo shoot quality has not been systematically examined. In this study, we investigate the environmental determinants of nutrient accumulation in Lei bamboo (*Phyllostachys violascens*) shoots by integrating environmental analysis, nutritional profiling, and transcriptomics. We identified soil organic matter, total porosity, and longitude as the primary factors influencing bamboo shoot nutrition, with higher soil organic matter correlating with enhanced nutrient content. Transcriptome analysis revealed that environmental conditions regulate key metabolic pathways, including starch metabolism (*e.g.*, *BGLU*, *SPS*) and flavonoid biosynthesis (*e.g.*, *PAL*, *4CL*), ultimately shaping bamboo shoot quality. Based on these findings, we developed a predictive model linking environmental factors, gene expression, and nutritional traits, providing a foundation for precision cultivation strategies. This study provides novel insights into plant-environment interactions governing bamboo shoot nutrition and offers actionable strategies for region-specific cultivation, aligning with consumer demand for healthier bamboo-based products.

## 1 Introduction

Bamboo shoots are a vital dietary component, valued for their rich proteins, vitamins, and fiber content, coupled with low calories value. However, their nutritional composition is highly dependent on environmental conditions, including soil properties, climate, and regional factors (Wang et al., 2021). Traditional studies have primarily focused on soil nutrient levels and their effects on bamboo shoot quality, yet these approaches offer limited insights into the underlying molecular mechanisms. While soil properties such as organic matter and nitrogen are known to influence bamboo shoot quality, the regulatory role of environmental factors on gene expression remains poorly understood.

Transcriptomics provides a powerful tool to bridge this gap by identifying differentially expressed genes (DEGs) and metabolic pathways associated with environmental conditions. Although transcriptomics has been widely used to study plant-environment interactions, its application to bamboo shoot quality regulation remains largely unexplored. This study integrates environmental analysis, nutritional profiling, and transcriptomics to investigate how soil factors influence gene expression and metabolic pathways in bamboo shoots, offering a molecular framework for precision cultivation strategies.

Among bamboo species, *Phyllostachys violascens* (Lei bamboo) is a high-quality, economically significant species endemic to China, primarily distributed in the hilly plains of Zhejiang and Jiangxi provinces. Known for its rapid growth, high yield, and strong market demand, Lei bamboo shoots are recognized as low-sugar, low-fat, high-protein, and high-fiber vegetables, rich in bioactive compounds with potential health benefits (Fan et al., 2023). However, variability in cultivars and yield inconsistencies pose significant challenges, arising from genetic differences, environmental factors, and management practices (Zhang et al., 2024a; Asante et al., 2024). Addressing these challenges requires a deeper understanding of the interactions between genetic, environmental, and management factors to optimize bamboo shoot production and quality.

Environmental variables, including temperature, light, humidity, rainfall, latitude, and altitude, significantly impact plant growth and nutrient accumulation. For instance, higher altitudes are associated with lower temperatures and increased ultraviolet (UV) radiation, which enhance the synthesis of secondary metabolites such as flavonoids and phenolics, thereby improving nutritional quality (Goud et al., 2022; Bhattacharya, 2022). Similarly, optimal temperatures (15-25°C) and adequate rainfall promote enzymatic activity and nutrient transport, essential for shoot tenderness and overall quality (Shi et al., 2020; Huang et al., 2022). Soil properties, including organic matter, nitrogen, phosphorus, and potassium, also play critical roles in bamboo growth. Well-drained, slightly acidic to neutral soils (pH 5.5-6.5) with high organic matter content are particularly beneficial, enhancing soil fertility and water retention (Lin et al., 2023; Chu et al., 2024).

Despite extensive research on environmental effects, there is a lack of comprehensive studies linking environmental conditions, molecular mechanisms, and nutritional traits in bamboo shoots. Specifically, it remains unclear how key environmental factors such as soil organic matter, total porosity, and geographical location regulate nutrient accumulation and bioactive compound synthesis. Furthermore, the molecular mechanisms underlying these effects, particularly the role of gene expression in metabolic pathways such as starch metabolism, cellulose synthesis, and flavonoid biosynthesis, are poorly understood.

This study aims to address these gaps by investigating the environmental drivers and transcriptomic regulation of nutritional variation in Lei bamboo shoots. We hypothesize that soil properties and geographical factors significantly influence nutritional composition by modulating gene expression in key metabolic pathways. The objectives of this study are twofold: (1) to identify key environmental variables affecting bamboo shoot nutrition, and (2) to uncover transcriptomic changes that regulate these traits. By integrating environmental data, transcriptomic analysis, and nutritional profiling, this study provides a comprehensive framework for optimizing bamboo shoot quality through precision cultivation and tailored regional management strategies.

## 2 Materials and methods

### 2.1 Sample collection area and environmental characteristics

**Figure 1:**
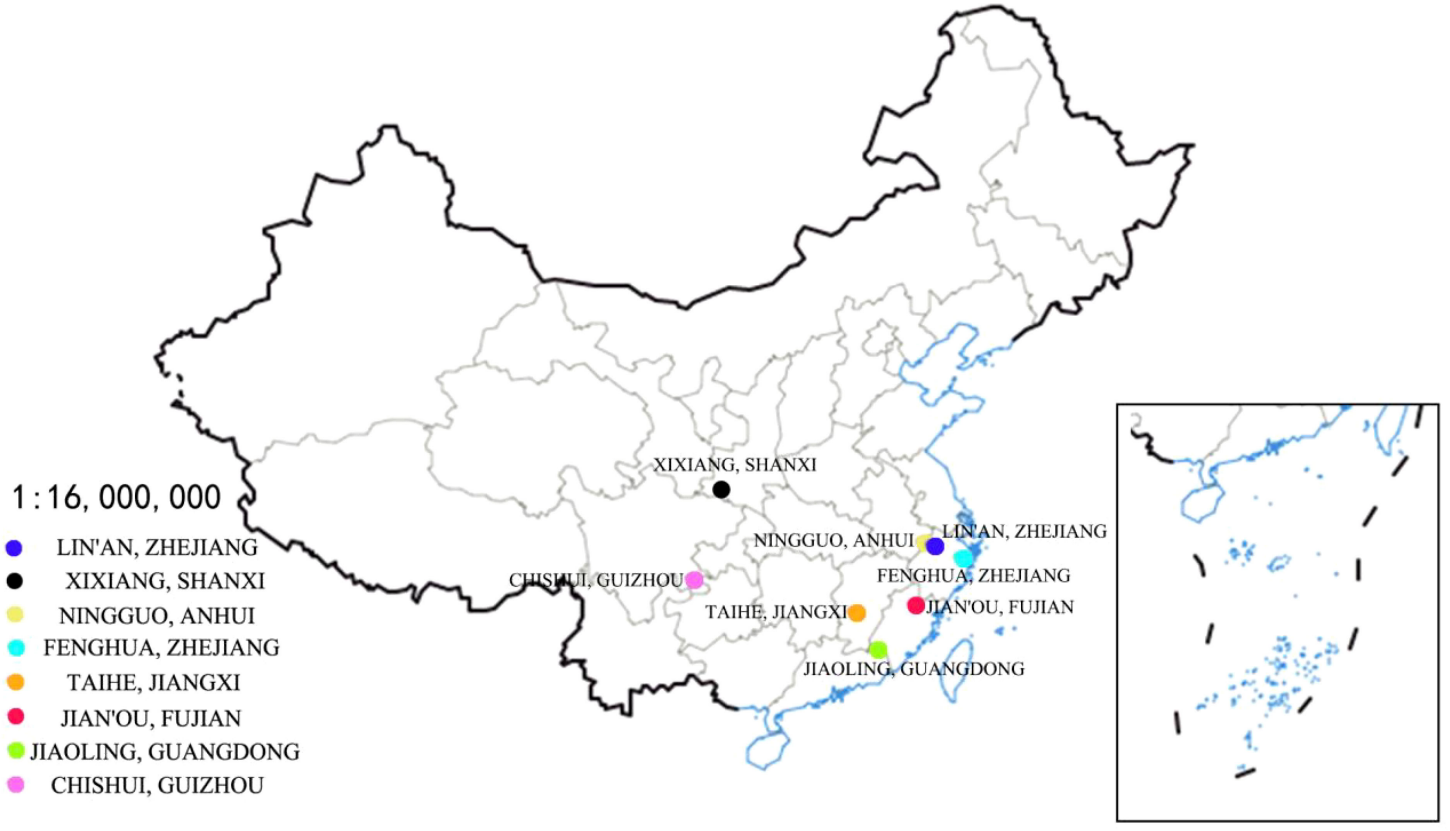
Study on the introduction distribution of bamboo shoots and the setting of sample land.

The natural distribution of *Phyllostachys violascens* was investigated by consulting local floras, relevant literature, and official media reports, in addition to field visits and surveys of bamboo industry developments across various regions. A comprehensive survey of the *P. violascens* germplasm resources throughout China was conducted. Soil and bamboo shoot samples from the same source (*P. violascens* from Lin’an, Zhejiang) were collected from eight regions: Lin’an (Zhejiang Province); Chishui (Guizhou Province); Fenghua (Zhejiang Province); Xixiang (Shaanxi Province); Taihe (Jiangxi Province); Jian’ou (Fujian Province); Jiaoling (Guangdong Province); and Ningguo (Anhui Province) (**Figure 1**; **Table 1**). These regions were selected because they represent the major cultivation areas of Lei bamboo in China and cover a wide range of climatic and geographical conditions, including variations in temperature, rainfall, and soil properties. Environmental factors including latitude, longitude, altitude, annual mean temperature, minimum and maximum temperatures, frost-free period, and annual precipitation were recorded for each cultivation area (**Table 2**).

**Table 1:** Information on the introduction of Lei bamboo (Xiye Lei bamboo) from Lin’an to various cultivation areas.

District	Longitude and latitude	Altitude (m)	Introduction year
Lin’an District, Hangzhou City, Zhejiang Province (LA)	119°33′E,30°17′N	173.08	*Phyllostachys violascens* ‘Xiye Lei bamboo’
Ningguo City, Xuancheng City, Anhui Province (NG)	119°14′E,30°23′N	213.60	2003
Fenghua District, Ningbo City, Zhejiang Province (FH)	121°15′E,29°38′N	158.58	2009
Jianou City, Nanping City, Fujian Province (JO)	118°41′E,27°14′N	150.61	1996
Taihe County, Ji’an City, Jiangxi Province (TH)	115°04′E,26°44′N	55.50	2010
Jiaoling County, Meizhou City, Guangdong Province (JL)	116°11′E,24°50′N	326.27	2017
Xixiang County, Hanzhong City, Shanxi Province (XX)	105°42′E,28°32′N	353.61	2014
Chishui, Zunyi City, Guizhou Province (CS)	107°12′E,33°15′N	552.62	2016

**Table 2:** Locations and climatic conditions of the study areas.

District	Average annual temperature(°C)	Minimum temperature (°C)	Maximum temperature (°C)	Frost-free period (days)	Annual precipitation (mm)
LA	16.45	-8	38	263.5	1628.6
NG	15.4	-10	39	226	1426.9
FH	16.3	-6	39.9	232	1475
JO	17	-7	40	275	1700
TH	19	-6	41.5	305	1457.9
JL	21.7	-0.6	39.8	250	1839.3
CS	18.1	-1.2	43.2	345	1195.7
XX	14.1	-9.3	37.6	245	850

LA, the Lin’an site, in Hangzhou City, Zhejiang Province; NG, the Ningguo site in Xuancheng City, Anhui Province; FH, the Fenghua site in Ningbo City, Zhejiang Province; JL, the Jiaoling site in Meizhou City, Guangdong Province. JO, the Jianou site in Nanping City, Fujian Province; TH, the Taihe site in Ji’an City, Jiangxi Province; CS, the Chishui site in Zunyi City, Guizhou Province; XX, the Xixiang site in Hanzhong City, Shanxi Province.

### 2.2 Soil sample collection and testing

Soil samples were collected in late March 2023 and again in late March to early April 2024 from the selected *P. violascens* introduction sites. The sampling period was chosen to coincide with the early growth stage of Lei bamboo shoots, when soil properties are most relevant to nutrient uptake and shoot development. To minimize the impact of seasonal variability, sampling was conducted under similar weather conditions (*e.g.*, temperature and rainfall) in both years, and historical data confirmed that March-April is a stable period for soil sampling in the study regions. In each cultivation area, three 5 × 5 m plots were established based on the following criteria: (1) the site had been cultivated with Lei bamboo for approximately ten years to ensure stable adaptation to local environmental conditions; (2) the plots were representative of the region’s typical soil and climate conditions; and (3) the plots had consistent management practices, such as fertilization and irrigation, to minimize confounding effects. From each plot, three soil samples were collected using a steel ring knife (soil sampler) along with three additional samples collected in soil bags. All samples were stored in ice packs and transported to the laboratory.

Soil samples taken with the ring knife were analyzed for physical properties, including bulk density (Landl et al., 2021), field capacity (Mao et al., 2022), and total porosity (Yang et al., 2019). A portion of the soil was air-dried, sieved through a 10-mesh sieve, and used to determine particle size partitioning (Ryżak and Bieganowski, 2011). Soil pH was analyzed following (Bottomley et al., 2020), while available phosphorus was measured according to (Yang et al., 2022), hydrolyzed nitrogen as described by (Cheng et al., 2023), and organic matter was determined using soil passed through a 100-mesh sieve, as outlined by (Te et al., 2022). Total nitrogen was quantified based on (Li et al., 2022), and total phosphorus was measured according to (Kozyrev et al., 2023). Additional soil metal element, including calcium, iron, potassium, magnesium, sodium and zinc, were analyzed using inductively coupled plasma optical emission spectrometry (ICP-OES, PerkinElmer Optima 8000). Calibration was performed using certified reference materials (CRM) for soil analysis, and control samples were included in each batch to ensure accuracy and precision.

### 2.3 Bamboo shoot collection and measurement

Bamboo shoot samples were collected from the *P. violascens* sites in late March to early April 2024. To ensure consistency in developmental stages, shoots were harvested at the same phenological stage across all regions, characterized by a shoot height of 20-30 cm and the presence of tightly closed sheaths. This stage represents a critical period for nutrient accumulation and metabolic activity and is widely recognized as the optimal harvest time for Lei bamboo shoots in commercial cultivation. In each region, three sample plots were selected, and three bamboo shoots were harvested from each plot. Fresh, similarly sized shoots, free from mechanical damage and pests, were selected for experimentation. The harvested shoots were immediately stored in ice packs, with nine shoots collected per region. In the laboratory, each shoot was cut into four equal parts lengthwise and one part was used as a sample. Three shoots were combined into a single sample, ground into powder using liquid nitrogen and stored at -80°C for the analysis of soluble sugars, starch, and cellulose (Li et al., 2020), along with the subsequent transcriptome analysis. Soluble proteins were quantified according to (Kučerová et al., 2019), while free amino acids, total phenols, and flavonoids were measured using the methods of (Bayram et al., 2021). Soluble tannins were determined following the method of (Kiewlicz and Rybicka, 2020). The remaining three-quarters of each shoot were placed in envelope bags, dried, and ground into powder using a ball mill for the determination of lignin content by an acetylation method.

### 2.4 Bamboo shoot transcriptome analysis

The bamboo shoot samples stored at -80°C were subjected to RNA extraction using the TRIzol method, followed by DNase I treatment to remove genomic DNA contamination. Three biological replicates per region were used, and each sample was sequenced in duplicate to ensure technical consistency. After RNA integrity assessment (RIN > 7.0) using an Agilent 2100 Bioanalyzer, libraries were prepared with the NEBNext Ultra RNA Library Prep Kit. Libraries that passed quality control were sequenced on an Illumina Novaseq 6000 platform (PE150). Adapter sequences and low-quality reads (Phred score < 20) were removed using Trimmomatic v0.39. Raw sequencing reads in FASTQ format were processed using FastQC (Chen et al., 2018) for quality assessment. Adapter sequences were removed using Trimmomatic v0.39 (Bolger et al., 2014) with default settings, followed by low-quality read filtering (Phred score < 20). Cleaned reads were then aligned to the Phyllostachys violascens reference genome using HISAT2 v2.2.1 (Kim et al., 2019) with default parameters. Reads with multiple mappings were discarded, and only uniquely mapped reads were retained for downstream analysis. Reads were aligned to the *Phyllostachys violascens* reference genome using HISAT2 v2.2.1 with default parameters. Differential gene expression (DEG) analysis was conducted using DESeq2 v1.30.0, with a significance threshold of |log_2_FC| ≥ 1 and an adjusted *P*-value < 0.05 (Benjamini-Hochberg correction). RNA-seq is widely accepted as a standalone tool for gene expression analysis in plant research (Ren et al., 2016; Liu et al., 2022), and numerous studies have successfully identified metabolic pathways without qRT-PCR validation. Although qRT-PCR validation was not conducted in this study, future research should incorporate qRT-PCR or proteomics to confirm transcriptomic findings, ensuring the robustness of gene expression patterns.

### 2.5 Statistical analysis

All statistical analyses were performed using SPSS v27.0 and R v4.2.3. Before conducting one-way ANOVA, normality was tested using the Shapiro-Wilk test (*P* > 0.05 indicates normal distribution), and homogeneity of variance was assessed using Levene’s test. If assumptions were violated, a non-parametric Kruskal-Wallis test was performed instead of ANOVA. *Post-hoc* multiple comparisons were conducted using Duncan’s multiple range test, and P-values were adjusted using the Bonferroni correction to minimize the risk of Type I errors. Multifactor ANOVA was conducted using DPS software (v7.05, China), and significant differences were determined using the Least Significant Difference (LSD) test, with an adjusted threshold of *P* < 0.05. Pearson’s correlation analysis was conducted to evaluate the relationships between environmental factors and soil properties. Mantel’s test was used to assess the correlation between soil and bamboo shoot traits, with Bray-Curtis distance applied for compositional data and Euclidean distance for continuous environmental variables. The coefficient of variation (*CV*) was calculated to quantify the variability of nutritional traits across different regions, defined as the ratio of the standard deviation to the mean value for each trait.

### 2.6 Data availability

The raw transcriptome and microbiome data have been uploaded to NCBl and can be retrieved using accession numbers PRJNA14042 and PRJNA1214209.

## 3 Results

### 3.1 Environmental differences across cultivation regions

The adaptability of Lei bamboo (*Phyllostachys violascens*) introduced from Lin’an (LA, the source region) to various cultivation regions was evaluated (**Table 2**). Lei bamboo shows distinct adaptability to specific geographic and climatic factors, including longitude (105°42´E-121°15´E), latitude (21°15´N-33°15´N), altitude (55.50-755.50 m), annual mean temperature (14.1-21.7°C), minimum temperature (−10°C), maximum temperature (43.2°C), frost-free period (226-345 days), and annual precipitation (850-1839.3 mm). As a native species of Lin’an, Lei bamboo was initially introduced within Zhejiang Province and gradually expanded from north to south. Each cultivation region underwent approximately ten years of adaptation to local climatic and geographic conditions, facilitating this study.

**Figure 2:**
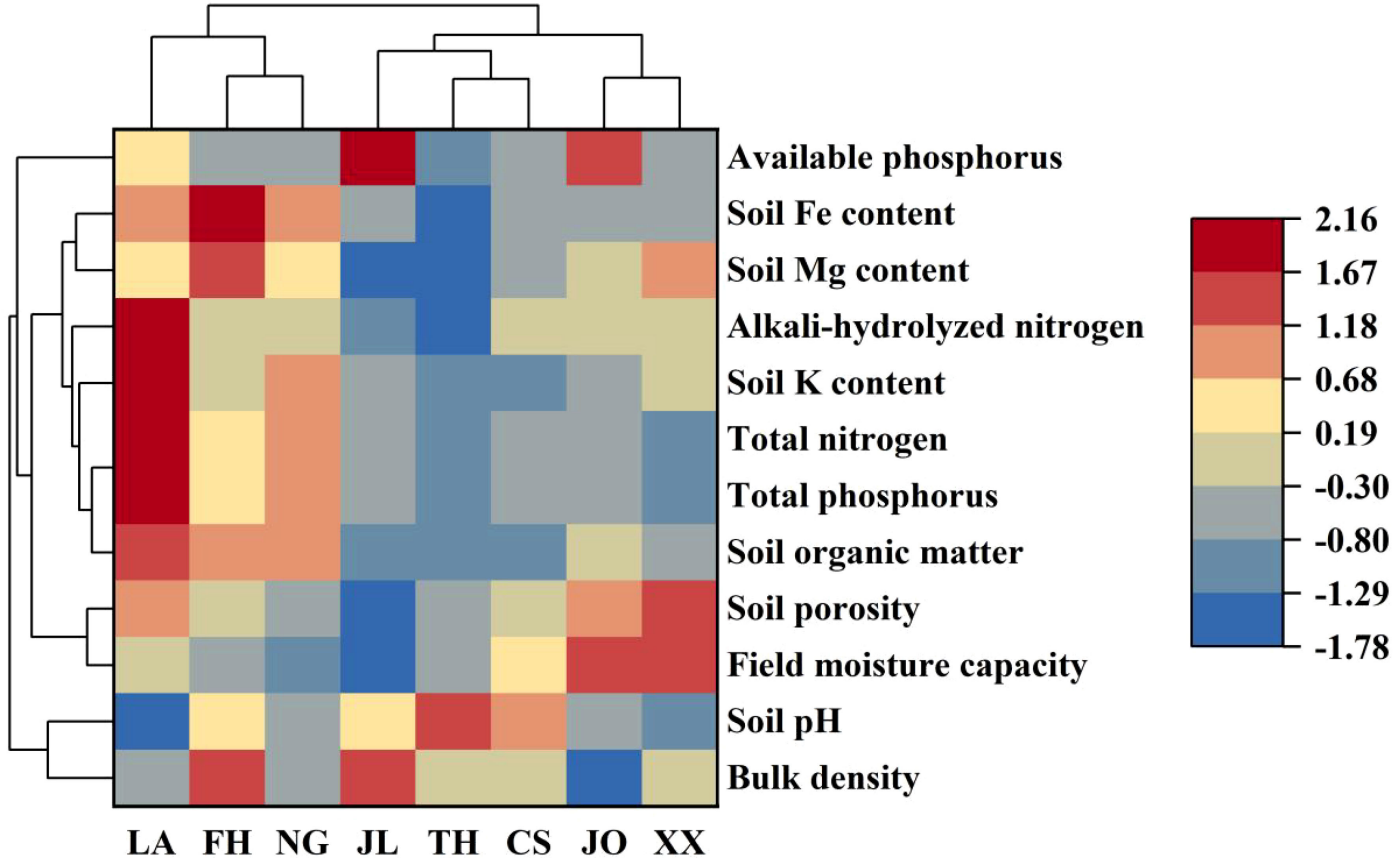
Clustered heatmap of soil properties across different cultivation regions of Lei bamboo. LA, soil samples from Lin ‘an; NG, soil samples from Ningguo; FH, soil samples from Fenghua; JL, soil samples from Jiaoling; JO, soil samples from Jian’ou; TH, soil samples from Taihe; CS, soil samples from Chishui; XX, soil samples from Xixiang.

Soil properties also significantly influence Lei bamboo growth. Lower soil bulk density and higher porosity enhance root aeration and bamboo growth. For instance, the soil bulk density in Jian’ou (1.07 g/cm³) was significantly lower than in other regions, and both Jian’ou and Xixiang exhibited higher field moisture capacity and total porosity (**Figure 2**). Lei bamboo thrives in slightly acidic to neutral soils, with an optimal pH range of 5.5-6.5. The soils in Ningguo and Jiaoling were most suitable for bamboo shoot growth based on their pH levels. Soil fertility, indicated by organic matter content, was significantly higher in Lin’an, Fenghua, and Ningguo compared to other regions. Jian’ou exhibited the highest total phosphorus content, while Jiaoling had the highest available phosphorus. Total nitrogen levels of 0.1%-0.2% were considered optimal for Lei bamboo growth, with Lin’an showing significantly higher levels of both total nitrogen and alkali-hydrolyzable nitrogen.Micronutrients in soil, such as magnesium (Mg), iron (Fe), and potassium (K) are vital for plant growth and soil health. Lin’an soil contained the highest potassium levels, while Fenghua soil had significantly higher magnesium and iron levels than other regions.

The soil characteristics of Lei bamboo cultivation regions exhibit distinct regional patterns, which are grouped into two main clusters based on their similarity. Cluster 1 (LA, FH, NG): These regions exhibit higher values for soil organic matter (106.58-92.75 g/kg), total phosphorus (4.53-3.12 g/kg), and soil K content (21.59-16.15 g/kg), indicating fertile soil conditions conducive to nutrient accumulation in bamboo shoots. Additionally, these regions have moderate soil porosity (49.36-32.86%) and field moisture capacity (33.48-19.08%), which support optimal root aeration and water retention.

Cluster 2 (JL, JO, TH, CS, XX): These regions are characterized by lower soil organic matter (26.31-37.42 g/kg) and total phosphorus (1.04-1.57 g/kg), but higher soil porosity (53.75-55.57%) and field moisture capacity (48.46-49.41%). Notably, Jian’ou (JO) and Xixiang (XX) stand out with the highest soil porosity and field moisture capacity, which may enhance root development and nutrient uptake despite lower soil fertility.These regional differences in soil properties highlight the importance of tailored soil management practices to optimize bamboo shoot quality. For example, regions with lower soil fertility (*e.g.*, JL, JO, TH) may benefit from organic amendments and targeted fertilization, while regions with higher fertility (*e.g.*, LA, FH, NG) may focus on maintaining soil structure and moisture levels.

### 3.2 Variations in nutritional traits of Lei bamboo shoots across regions

**Figure 3:**
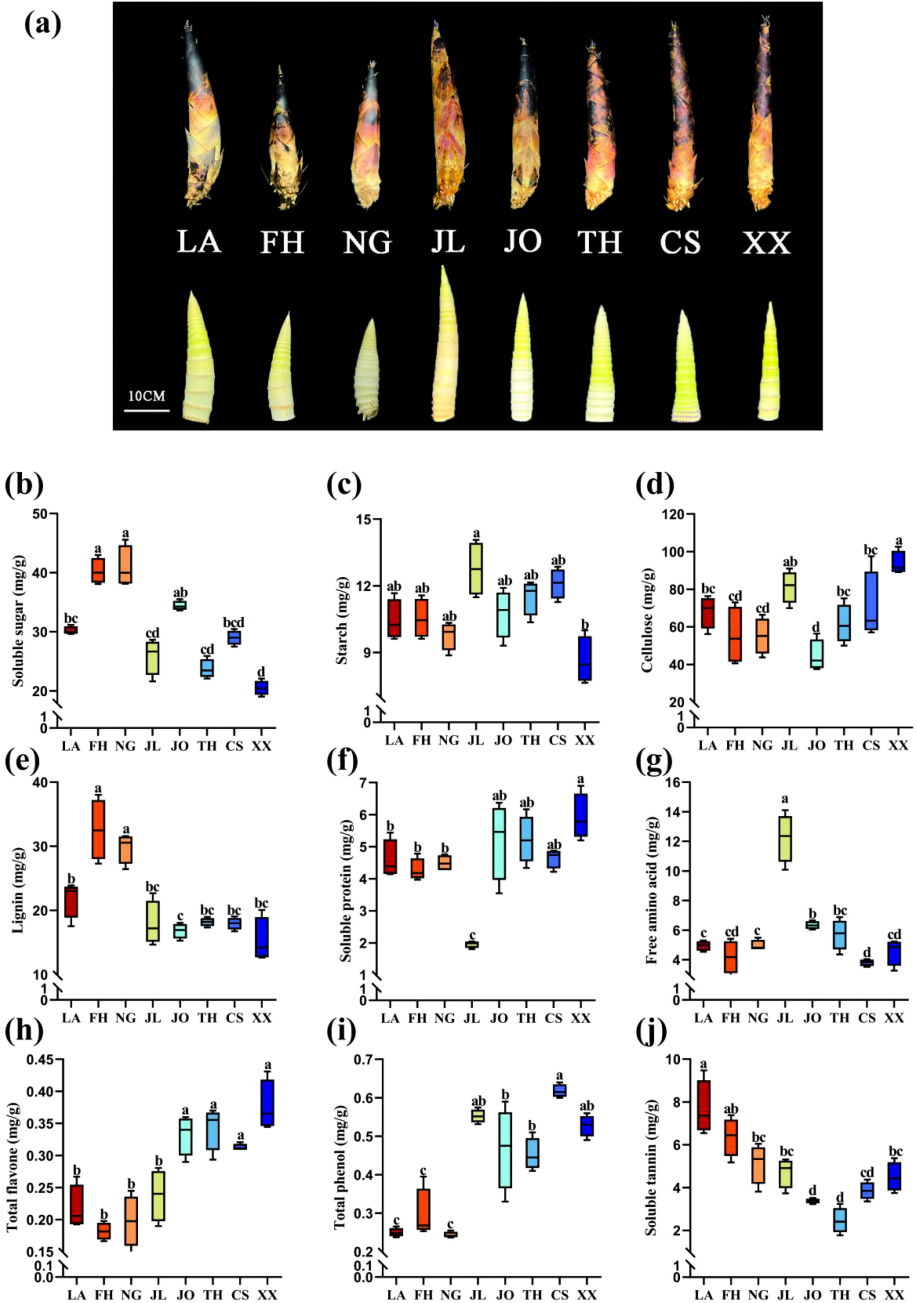
Apparent and nutritional characters of bamboo shoots. **(A)** Comparison of shell-contained and shell-removed images of bamboo shoots collected from different areas of bamboo cultivation. **(B-J)** Nutritional properties of Lei bamboo bamboo shoots (LA) transplanted to different cultivation regions. LA, bamboo shoot samples from Lin ‘an; NG, bamboo shoot samples from Ningguo; FH, bamboo shoot samples from Fenghua; JL, bamboo shoot samples from Jiaoling; JO, bamboo shoot samples from Jian’ou; TH, bamboo shoot samples from Taihe; CS, bamboo shoot samples from Chishui; XX, bamboo shoot samples from Xixiang. Different lowercase letters (a, b, c, d) above the bars indicate significant differences (p < 0.05) in the nutritional quality of bamboo shoots among different sample sites, as determined by one-way ANOVA followed by Tukey's post hoc test.

The appearance and nutritional traits of Lei bamboo shoots, including soluble sugars, starch, cellulose, lignin, soluble protein, free amino acids, total flavonoids, total phenols, and soluble tannins, were analyzed for both the source and introduction regions. Significant differences were observed across all traits. Bamboo shoots from the Lin’an provenance area were remarkably robust than those from other introduced regions (**Figure 3A**). Bamboo shoots from Jiaoling, Taihe, Xixiang and other regions were slenderer. Key nutritional components, such as total sugars, starch, cellulose, and lignin, exhibited distinct patterns. Xixiang bamboo shoots had the lowest total sugar and starch levels (**Figures 3B, C**), approximately 70% of those in Lin’an, while cellulose content was 1.4 times higher than in Lin’an (**Figure 3D**). Jian’ou bamboo shoots had the lowest lignin content, approximately 70% of that in Lin’an (**Figure 3E**).

Further variations were observed in soluble proteins, free amino acids, and secondary metabolites (total flavonoids, total phenols, and tannins). Xixiang bamboo shoots had 1.4 times more soluble protein than those from Lin’an (**Figure 3F**). Jiaoling exhibited the highest free amino acid content, 3.2 times that of Chishui and 1.3 times that of Lin’an (**Figure 3G**). Fenghua bamboo shoots had the lowest total flavonoid levels, only 80% of those in Lin’an (**Figure 3H**). Total phenol content was lowest in Ningguo, while tannin content in Taihe was only 34% of that in Lin’an (**Figures 3I, J**).

### 3.3 Correlations between nutritional traits and environmental factors

**Figure 4:**
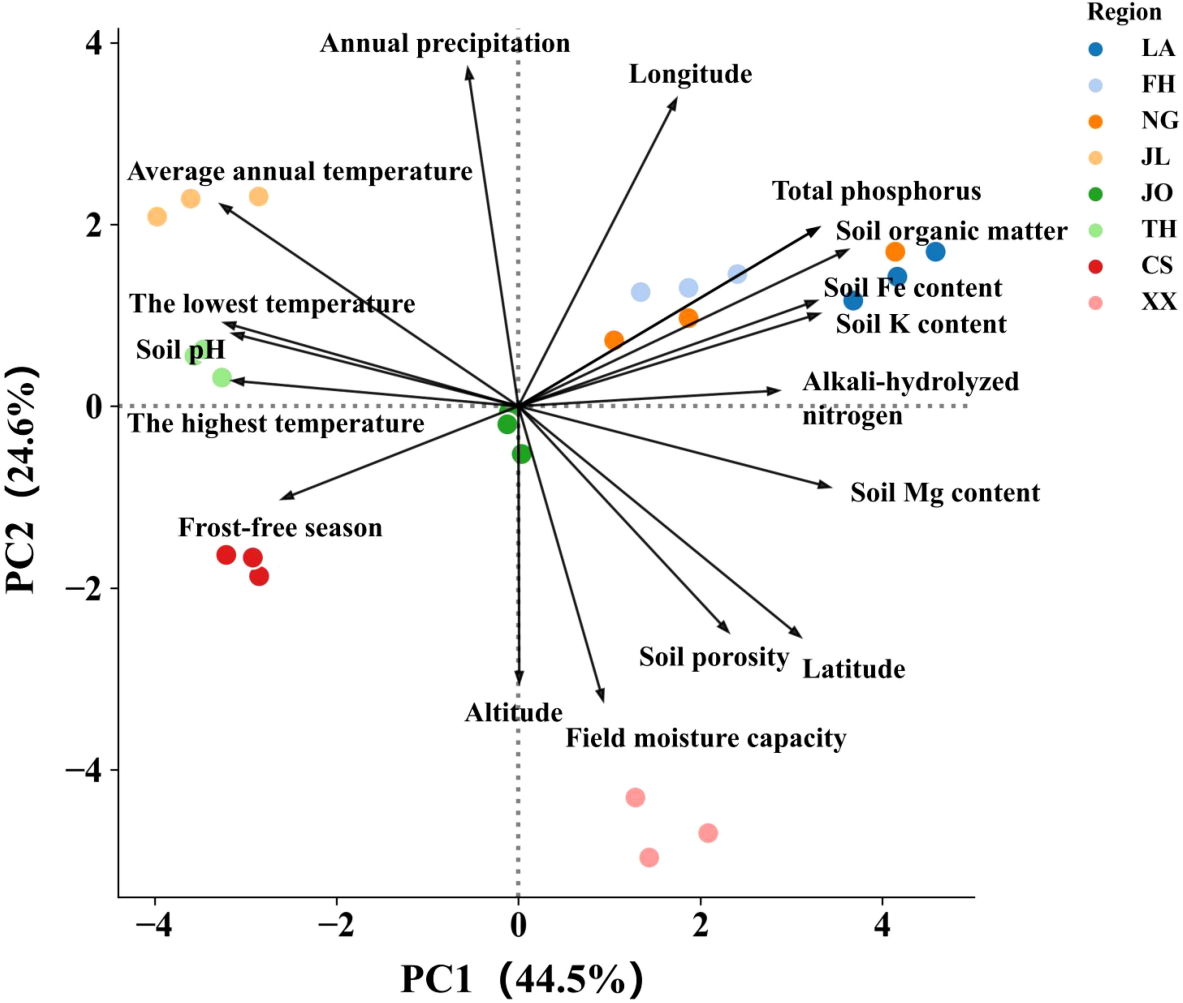
Principal Component Analysis (PCA) of soil environmental factors and nutritional traits in Lei bamboo shoots.

Principal Component Analysis (PCA) was performed to identify the key environmental factors influencing the nutritional quality of Lei bamboo shoots (**Figure 4**). The first two principal components (PC1 and PC2) explained 69.05% of the total variance, with PC1 contributing 44.47% and PC2 contributing 24.58%. The loading values of each variable on PC1 and PC2, as well as their total importance, are summarized in **Figure 4**. The most influential variables on PC1 were soil organic matter (loading = 0.878), total phosphorus (loading = 0.799), total nitrogen (loading = 0.799), and soil Mg content (loading = 0.827). These variables are strongly associated with soil fertility and nutrient availability, indicating that soil quality is a major driver of bamboo shoot nutritional quality.

Latitude (loading = 0.752) and average annual temperature (loading = -0.793) also showed significant contributions to PC1, suggesting that geographical and climatic factors play a critical role in shaping nutrient accumulation in bamboo shoots.The most influential variables on PC2 were annual precipitation (loading = 0.900), longitude (loading = 0.819), and soil porosity (loading = -0.599). These variables highlight the importance of water availability and soil structure in regulating bamboo shoot nutrition.

Field moisture capacity (loading = -0.781) and altitude (loading = -0.731) also contributed significantly to PC2, further emphasizing the role of environmental conditions in nutrient uptake and metabolic processes.

The PCA analysis revealed that soil fertility (organic matter, phosphorus, nitrogen), geographical and climatic factors (latitude, temperature, precipitation), and soil structure (porosity, moisture capacity) are the primary drivers of Lei bamboo shoot nutritional quality. These findings provide a scientific basis for optimizing bamboo cultivation practices through targeted soil management and region-specific strategies.

The coefficients of variation (*CV*) for the nutritional traits of Lei bamboo shoots ranged from 22.96% to 36.21%, indicating significant variability across different cultivation regions. The highest CV was observed for free amino acid content (36.21%), followed by lignin (34.63%) and total phenolics (34.41%). These results suggest that free amino acids, lignin, and total phenolics are the most variable nutritional traits in Lei bamboo shoots, while starch content (CV = 22.96%) and total flavonoids (CV = 24.93%) showed relatively lower variability (**Table 3**).

**Table 3:** Total variation of nutritional characters of bamboo shoots in provenance and introduced areas.

Nutritional characters of bamboo shoots	Mean ± SD	Maximum	Minimum	CV%
Soluble sugar	30.8 ± 8.06	48.13	48.13	26.18
Starch	11.29 ± 2.59	16.76	16.76	22.96
Cellulose	62.97 ± 17.88	92.62	92.62	28.39
Lignin	22.48 ± 7.79	38.03	38.03	34.63
Soluble protein	27.19 ± 7.13	38.2	38.2	26.23
Free amino acid	150.95 ± 54.66	285.57	285.57	36.21
Total flavone	0.28 ± 0.07	0.4	0.4	24.93
Total phenol	0.43 ± 0.15	0.64	0.64	34.41
Soluble tannin	4.72 ± 1.47	7.65	7.65	31.13

### 3.4 Transcriptome analysis of Lei bamboo shoots across cultivation regions

**Figure 5:**
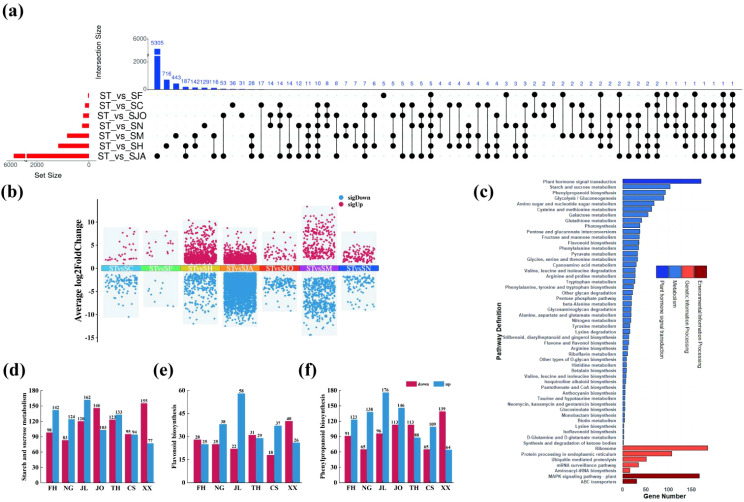
The original number and intersection number of transcriptomic genes in each group of bamboo shoots in different cultivation areas. ST, the transcriptome samples of bamboo shoots from Lin ‘an; SF, the transcriptome samples of bamboo shoots from Fenghua; SN, the transcriptome samples of bamboo shoots from Ningguo; SM, the transcriptome samples of bamboo shoots from Jiaoling; SJO, the samples transcriptome of bamboo shoots from Jian’ou; SJA, the transcriptome samples of bamboo shoots from Taihe; SC, the transcriptome samples of bamboo shoots from Chishui; SH, the transcriptome samples of bamboo shoots from Xixiang. LA, bamboo shoot samples from Lin ‘an; NG, bamboo shoot samples from Ningguo; FH, bamboo shoot samples from Fenghua; JL, bamboo shoot samples from Jiaoling; JO, bamboo shoot samples from Jian’ou; TH, bamboo shoot samples from Taihe; CS, bamboo shoot samples from Chishui; XX, bamboo shoot samples from Xixiang. B-E, Kyoto Encyclopedia of Genes and Genomes (KEGG) enrichment analysis of differential genes in bamboo shoots from different introduced distribution areas.

Transcriptome data of Lei bamboo shoots from different regions were compared with those from the original source in Lin’an. Differentially expressed genes (DEGs) were identified (**Figure 5A**), revealing significant variations in gene expression patterns across regions. The number of DEGs was as follows: 5305 in the ST vs. SJA group, 716 in the ST vs. SH group, 443 in the ST vs. SM group, 129 in the ST vs. SN group, 36 in the ST vs. SC group, 31 in the ST vs. SJO group, and 5 in the ST vs. SF group. These DEGs exhibited region-specific trends in expression (**Figure 5B**), with the ST vs. SJA group showing the highest number of DEGs (5835), predominantly down-regulated (4746 down-regulated vs. 1089 up-regulated).

To explore the biological relevance of the identified DEGs, KEGG pathway analysis was performed on 2819 DEGs, revealing 133 enriched pathways (**Figure 5C**). Key metabolic pathways critical for the nutritional composition of Lei bamboo shoots were identified, including starch and sucrose metabolism, phenylpropanoid biosynthesis, and flavonoid biosynthesis. These pathways are known to regulate the biosynthesis of major nutritional compounds (*e.g.*, sugars, starches, proteins, amino acids) and secondary metabolites (*e.g.*, flavonoids, phenolics, and tannins), which contribute to the nutritional and medicinal properties of bamboo shoots (Gong et al., 2024).

#### 3.4.1 Starch and sucrose metabolism pathway

Genes involved in starch and sucrose metabolism were enriched in multiple regions, indicating their role in plant growth, energy supply, and adaptation to environmental conditions (**Figure 5D**). The number of DEGs observed in different comparisons includes 240 in ST vs. SF (142 up-regulated), 207 in ST vs. SN (124 up-regulated), 282 in ST vs. SM (162 up-regulated), 249 in ST vs. SJO (146 down-regulated), 256 in ST vs. SJA (133 up-regulated), 189 in ST vs. SC (95 down-regulated), and 232 in ST vs. SH (155 down-regulated).

**Figure 6:**
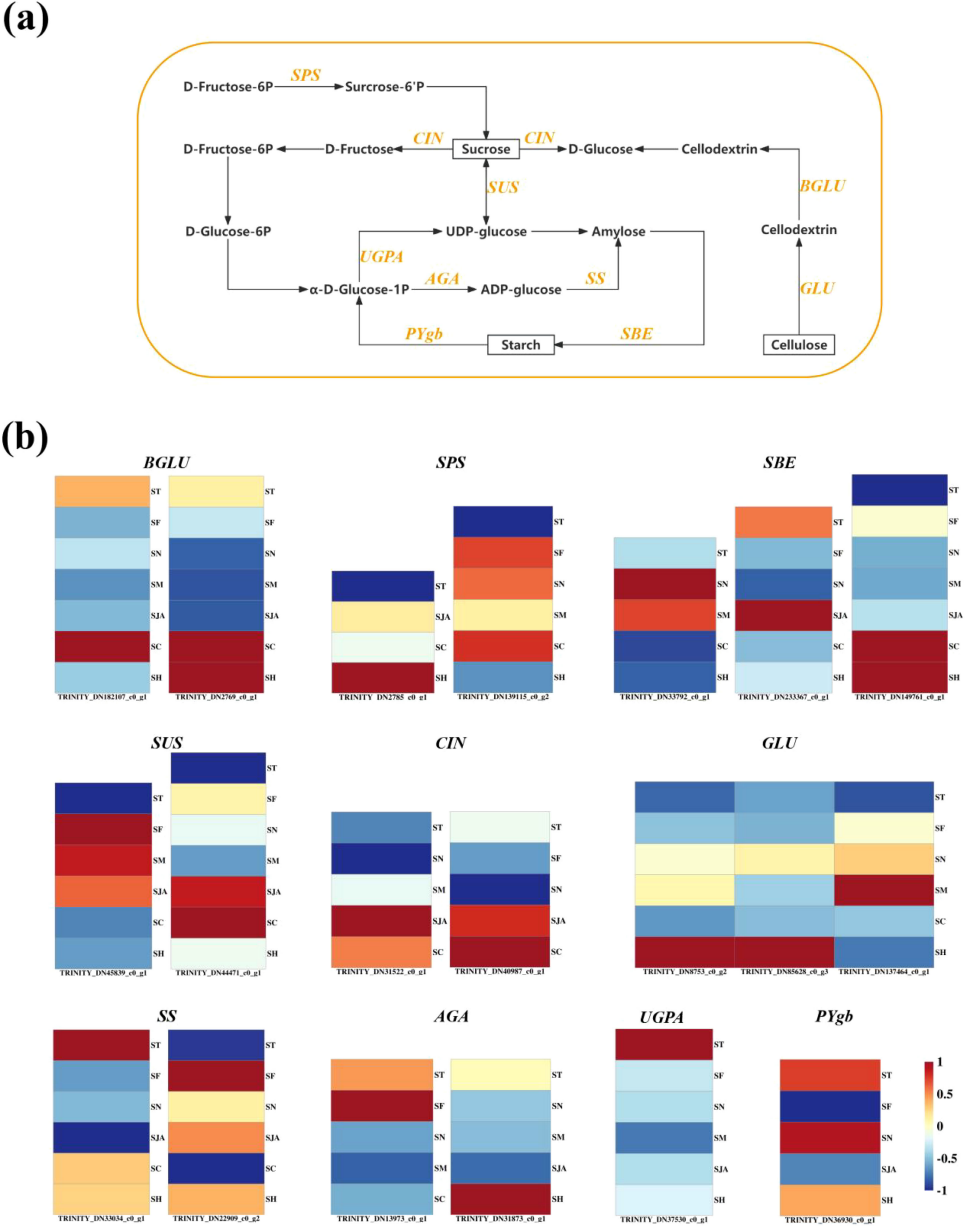
Transcriptome regulation of starch and sucrose metabolism in bamboo shoots. **(A)** Metabolic pathways of starch and sucrose in bamboo shoots. **(B)** DEGs related to starch and sucrose metabolism in Lei bamboo shoots. Heat map was constructed based on FPKM values, and rows and columns indicate DEGs and samples, respectively. The color scale represents the Z-score calculated. BGLU, Beta-glucosidase; SPS, Sucrose Phosphate Synthase; SBE, Starch Branching Enzyme; SUS, Sucrose Synthase;CIN, Cinnamyl Alcohol Dehydrogenase; GLU, Glucose; SS, Sucrose Synthase; AGA, Agarose; UGPA, UDP-Glucose Pyrophosphorylase; PYgb, Glycogen Phosphorylase B; ST, the transcriptome samples of bamboo shoots from Lin ‘an; SF, the transcriptome samples of bamboo shoots from Fenghua; SN, the transcriptome samples of bamboo shoots from Ningguo; SM, the transcriptome samples of bamboo shoots from Jiaoling; SJO, the samples transcriptome of bamboo shoots from Jian’ou; SJA, the transcriptome samples of bamboo shoots from Taihe; SC, the transcriptome samples of bamboo shoots from Chishui; SH, the transcriptome samples of bamboo shoots from Xixiang.

Key genes, such as *BGLU* (*e.g.*, TRINITY_DN182107_c0_g1), *SPS* (*e.g.*, TRINITY_DN2785_c0_g1), *SBE* (*e.g.*, TRINITY_DN33792_c0_g1), *SUS* (*e.g.*, TRINITY_DN45839_c0_g1), *CIN*, *GLU*, *SS, AGA*, *UGPA*, and *PYgb*, were significantly enriched in the starch and sucrose metabolism pathway (**Figure 6A**). The expression of these genes varied across regions, with *BGLU* expression lower in bamboo shoots from Taihe and Jiaoling compared to Lin’an, while Chishui showed the highest expression (1.65-fold higher) (**Figure 6B**). Conversely, *SPS* expression was higher in Fenghua and Ningguo, with Lin’an exhibiting the lowest expression (0.05). *SBE* expression was highest in Jiaoling bamboo shoots, and *SUS* gene expression was elevated in Fenghua and Ningguo. Interestingly, *GLU* gene expression was 3.4 times higher in Fenghua bamboo shoots than in Lin’an. These findings align with previous studies demonstrating the role of starch and sucrose metabolism in regulating carbohydrate storage and energy balance in bamboo (Wang et al., 2022).

#### 3.4.2 Phenylpropanoid biosynthesis pathway

**Figure 7:**
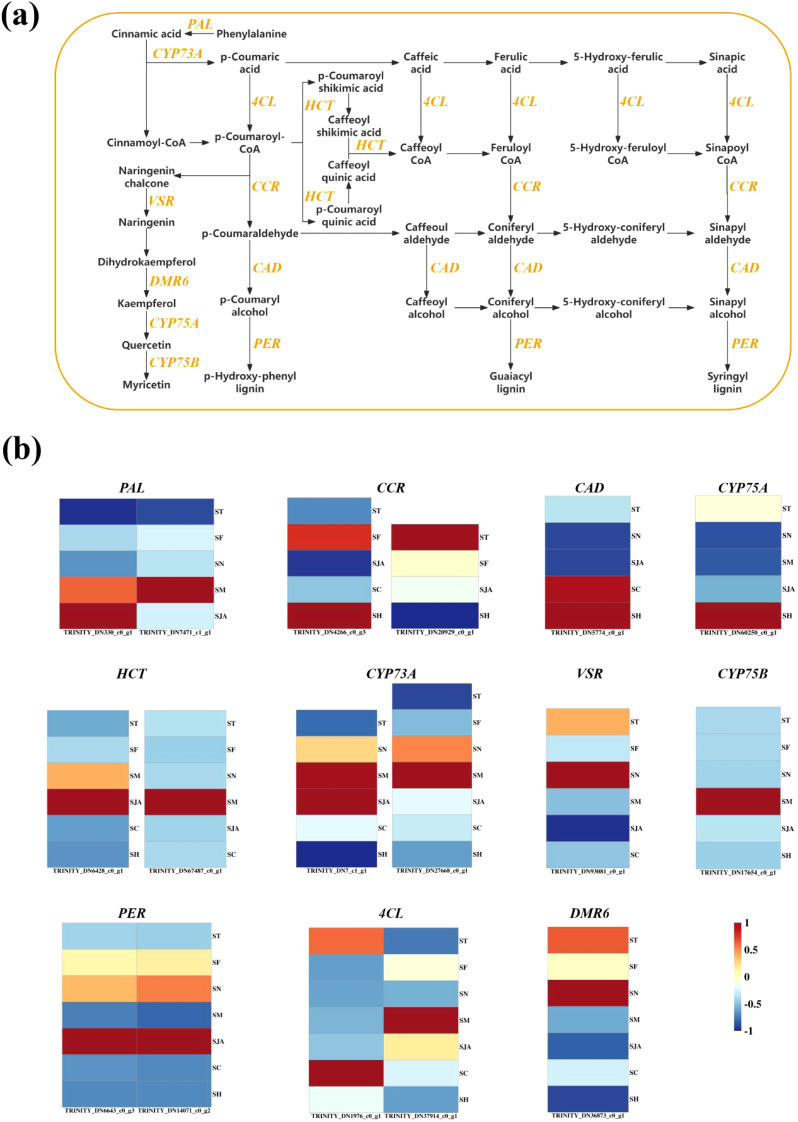
Transcriptome regulation of Phenylpropanoid biosynthesis and Flavonoid biosynthesis in bamboo shoots. **(A)** Phenylpropanoid biosynthesis and Flavonoid biosynthesis in bamboo shoots. **(B)** DEGs related to Phenylpropanoid biosynthesis and Flavonoid biosynthesis in Lei bamboo shoots. Heat map was constructed based on FPKM values, and rows and columns indicate DEGs and samples, respectively. The color scale represents the Z-score calculated. PAL, Phenylalanine Ammonia-Lyase; CYP73A, Cinnamate-4-Hydroxylase; 4CL, 4-Coumarate-CoA Ligase; HCT, Hydroxycinnamoyl-CoA; CCR, Cinnamate-CoA Reductase; VSR, Vesicle-Associated Membrane Protein (VAMP)-Related; CAD, Cinnamyl Alcohol Dehydrogenase; PER,Peroxidase; DMR6, Defective in Mediation of Resistance 6; CYP75A, Flavonoid 3’-Hydroxylase; CYP75B, Flavonoid 3’,5’-Hydroxylase; ST, the transcriptome samples of bamboo shoots from Lin ‘an; SF, the transcriptome samples of bamboo shoots from Fenghua; SN, the transcriptome samples of bamboo shoots from Ningguo; SM, the transcriptome samples of bamboo shoots from Jiaoling; SJO, the samples transcriptome of bamboo shoots from Jian’ou; SJA, the transcriptome samples of bamboo shoots from Taihe; SC, the transcriptome samples of bamboo shoots from Chishui; SH, the transcriptome samples of bamboo shoots from Xixiang.

This pathway is essential for the structural integrity and stress resistance of bamboo shoots (**Figure 5F**). Notable DEGs involved in this pathway include *PAL* (*e.g.*, TRINITY_DN330_c0_g1), *CCR* (*e.g.*, TRINITY_DN4266_c0_g3), *CAD*, *HCT*, *CYP73A*, *PER*, and *4CL* (**Figure 7A**). In particular, *PAL* catalyzes the conversion of phenylalanine to cinnamic acid, a precursor to lignin and flavonoids. *CCR* and *4CL* are involved in lignin biosynthesis, while *CAD* and *HCT* contribute significantly to the synthesis of lignin.

The expression of *PAL* in Jiaoling bamboo shoots was 8-fold higher than in Lin’an, and *4CL* expression was 6-fold higher (**Figure 7B**). In contrast, *CCR* gene expression was lower in Taihe bamboo shoots than in Lin’an. *CYP73A* expression was higher in Fenghua and Ningguo, while *PER* expression was elevated in Chishui and Xixiang compared to Lin’an. These results are consistent with previous studies highlighting the role of phenylpropanoid biosynthesis in lignin and flavonoid production, which are critical for plant defense and structural support (Dong and Lin, 2021).

#### 3.4.3 Flavonoid biosynthesis pathway

Flavonoids, which contribute to the nutritional and medicinal properties of bamboo shoots, are synthesized via this metabolic pathway (**Figure 5E**). DEGs enriched in this pathway include *VSR*, *DMR6*, *CYP75A*, and *CYP75B*. *VSR* is involved in secondary metabolite transport, and *DMR6* is linked to plant resistance and secondary metabolite synthesis. *CYP75A* and *CYP75B* play key roles in flavonoid biosynthesis, particularly the synthesis of myricetin.

Expression of *VSR* and *DMR6* was lower in Fenghua bamboo shoots compared to Lin’an, while *CYP75A* expression was highest in Xixiang bamboo shoots (1.54-fold higher). *CYP75B* expression was highest in Zhuoling (18.74), while Ningguo exhibited lower expression compared to Lin’an, and Xixiang showed higher levels than Lin’an. These findings corroborate previous research demonstrating the role of flavonoids in plant stress responses and nutritional quality (Li et al., 2023c).

This study builds upon previous research on bamboo nutrition by providing a comprehensive transcriptomic analysis of Lei bamboo shoots across multiple cultivation regions. While earlier studies have explored the genetic regulation of nutrient uptake and secondary metabolite biosynthesis in bamboo (Hou et al., 2022), our work extends these findings by identifying region-specific DEGs and their associated metabolic pathways. For example, the significant enrichment of starch and sucrose metabolism genes in Fenghua and Ningguo aligns with previous reports on the role of these pathways in carbohydrate storage and energy balance (Li et al., 2023a). Additionally, the elevated expression of phenylpropanoid biosynthesis genes in Jiaoling supports earlier findings on the importance of lignin and flavonoid production in plant defense and structural integrity (Dong and Lin, 2021).

This study advances our understanding of the transcriptomic regulation of bamboo shoot traits by identifying key genes and pathways associated with nutritional composition and stress responses. For instance, the region-specific expression of *BGLU*, *SPS*, and *PAL* genes provides new insights into the genetic mechanisms underlying carbohydrate metabolism and secondary metabolite biosynthesis. These findings complement previous research on the transcriptomic regulation of bamboo shoot development and stress responses (Shou et al., 2020), offering a more comprehensive understanding of the genetic basis of bamboo nutrition.

### 3.5 Correlation analysis between nutritional traits of Lei bamboo shoots and environmental factors

**Figure 8:**
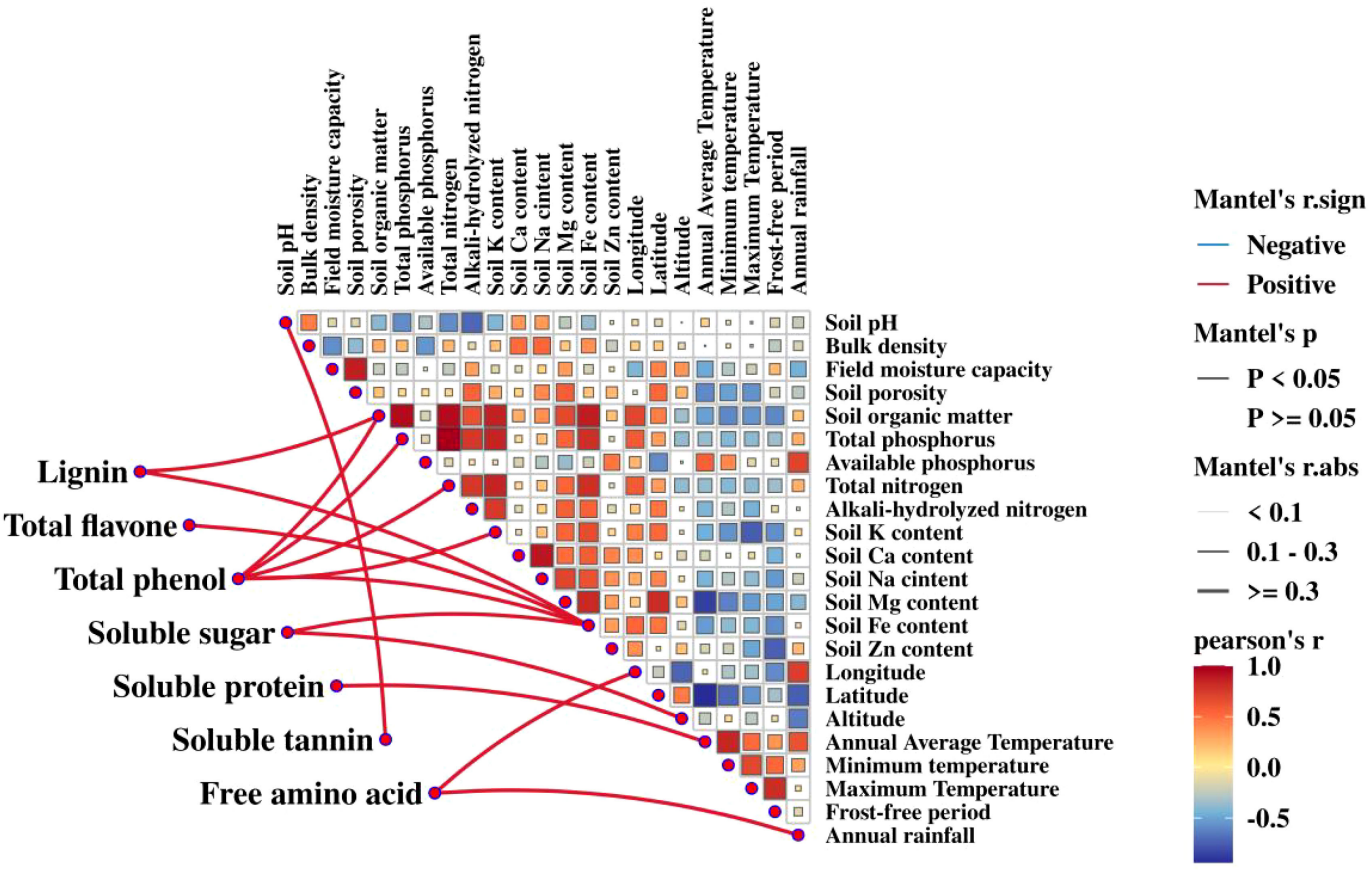
Correlation analysis between the nutritional qualities of Lei bamboo shoots and environmental factors across cultivation sites.

The correlation analysis revealed significant relationships between the nutritional traits of Lei bamboo shoots and environmental factors across various cultivation regions (**Figure 8**). Strong correlations were found between soil properties and key nutritional traits of the bamboo shoots. Soil organic matter content exhibited high positive correlations with total phosphorus, nitrogen, potassium, and iron content, suggesting that the nutrient composition of the soil plays a crucial role in determining bamboo shoot nutrition. Additionally, Soil field moisture capacity was positively correlated with total porosity, highlighting the importance of soil structure in nutrient retention.

Environmental factors such as temperature and latitude were also critical in shaping the nutritional profile of bamboo shoots. For instance, soluble protein content in bamboo shoots was positively correlated with annual average temperature, while magnesium levels showed a negative correlation with temperature but a positive correlation with latitude and iron content. These findings indicate that temperature and geographical factors influence nutrient availability and incorporation into bamboo shoots.

Specific nutritional compounds also demonstrated strong correlations with both environmental and soil factors. Lignin content was positively correlated with both soil organic matter and iron content, reinforcing the role of soil quality in lignin synthesis. Free amino acids, essential for bamboo growth, were significantly correlated with longitude and annual rainfall, suggesting that geographic location and rainfall patterns affect amino acid composition. Furthermore, total phenolics, which have antioxidant properties, were strongly correlated with soil organic matter, total phosphorus, and nitrogen, underscoring the importance of soil nutrient composition in phenolic accumulation.

The Mantel test revealed significant positive correlations between certain environmental factors and biochemical compounds, particularly involving soil organic matter, soil Fe content, and specific climatic variables. These findings underscore the intricate relationships between soil properties, climatic conditions, and biochemical composition, providing valuable insights for ecological and agricultural research.

## 4 Discussion

### 4.1 Differences in environmental factors between the source and transplanted regions and identification of key drivers

The nutritional traits of Lei bamboo shoots are shaped by a complex interplay of environmental factors, including soil properties, climate conditions, and geographical location (Aribal et al., 2022; Chen et al., 2024; Zuo et al., 2023). Key drivers such as soil organic matter, total porosity, and longitude significantly influence nutrient accumulation (Sun et al., 2020), with regions like Lin’an showing enhanced soluble sugars and starch due to high organic matter and optimal temperatures, while Xixiang exhibited elevated cellulose content under cooler conditions. Longitude mediated its effects through climate variations, with eastern regions benefiting from higher rainfall for amino acid synthesis and western regions producing more phenolic compounds due to increased solar radiation. These findings align with previous research (Zhang et al., 2020; Zheng et al., 2022; Wu et al., 2024) and highlight the potential for precision agriculture to optimize bamboo shoot quality through region-specific cultivation practices.

However, this study has limitations that warrant consideration. The frequency of sampling (once per year) may not fully capture seasonal fluctuations in soil conditions, which could influence nutrient dynamics. To improve reliability, future studies should incorporate multiple sampling periods, such as pre- and post-harvest, to better account for temporal variability. Additionally, this study does not address the potential impact of climate change or the role of the soil microbiome. Rising temperatures and altered rainfall patterns could significantly affect nutrient accumulation, while microbial communities likely play a crucial role in nutrient cycling and plant-microbe interactions (Wahid et al., 2020). Future research should integrate climate change scenarios and soil microbiome analysis to provide a more comprehensive understanding of bamboo nutrition.

In conclusion, this study offers novel insights into the environmental and molecular mechanisms governing bamboo shoot nutrition. By identifying key environmental drivers and transcriptomic changes, we provide a scientific foundation for optimizing bamboo shoot quality through precision cultivation and sustainable management strategies. These findings not only advance our understanding of plant-environment interactions but also support the development of nutrient-rich bamboo-based products to meet growing consumer demand (Acharya et al., 2023; Zhang et al., 2024b).

### 4.2 Trait changes in bamboo shoots driven by environmental factors

Nutritional compounds in plants are often stored as non-structural carbohydrates, such as soluble sugars and starch, which are essential for plant development (Dobbelstein et al., 2019; Zhang et al., 2021). In bamboo shoots, soluble sugars, starch, cellulose, and lignin serve as key nutritional indicators and are significantly influenced by soil and environmental conditions (Tang et al., 2022; Zhang et al., 2024b; Ma et al., 2024). The results of this study showed that bamboo shoots from Xixiang had the highest cellulose content and the lowest levels of total sugar and starch. This was likely due to the region’s high soil porosity, cooler temperatures, and adequate water supply, which might have favored cellulose synthesis while limiting starch accumulation. In contrast, bamboo shoots from Jian’ou exhibited the lowest lignin content, likely attributable to the region’s optimal pH and high phosphorus availability, which improved shoot quality (Bindraban et al., 2020). Moreover, the study found that free amino acid content positively correlated with longitude, indicating that regions with high precipitation levels promoted amino acid synthesis, thereby improving the nutritional value of bamboo shoots. Other key soil nutrients, such as potassium and iron, were also critical in shaping the nutritional composition of bamboo shoots. Potassium was positively correlated with soluble sugar, while iron was linked with lignin content, suggesting a direct influence of these nutrients on the primary metabolic pathways in bamboo shoots.

Secondary metabolites such as total phenolics, flavonoids, and tannins contribute to the characteristic bitterness of bamboo shoots (Gong et al., 2024; Wang et al., 2023). Higher total phenolic content in bamboo shoots from Jian’ou and Chishui was associated with high soil organic matter content and phosphorus levels. Conversely, the lowest flavonoid content was observed in Fenghua, where low soil iron content likely suppressed flavonoid biosynthesis. Taihe exhibited the lowest tannin content, which could be linked to soil pH and organic matter content. These findings emphasize the complex regulation of both primary and secondary metabolites by environmental factors, leading to significant regional variations in bamboo shoot nutrition. The results provide a scientific foundation for region-specific management and precision cultivation practices to enhance bamboo shoot quality.

### 4.3 Transcriptome variations and their regulation of nutritional traits

Transcriptome analysis revealed that environmental conditions, particularly soil organic matter and nitrogen levels, significantly influence gene expression in key metabolic pathways, such as starch metabolism, cellulose synthesis, and flavonoid biosynthesis. For instance, low soil organic matter and nitrogen levels in Jiaoling, Taihe, and Xixiang down-regulated genes like *BGLU*, *AGA*, *PYgb*, and *UGPA*, which are involved in starch and sucrose metabolism, potentially explaining the reduced sugar accumulation in these regions. Conversely, the upregulation of *PER* (Peroxidase) in Jian’ou correlated with higher fiber content, while *CYP75A* overexpression in Jiaoling aligned with elevated flavonoid levels. These findings highlight the molecular mechanisms underlying regional variations in bamboo shoot quality.

The regulation of these metabolic pathways is not limited to individual genes but involves broader transcriptional networks. Transcription factors (TFs) such as *NAC*, *MYB*, and *WRKY* are known to modulate starch metabolism, phenylpropanoid biosynthesis, and flavonoid pathways in response to environmental stressors (Zhang et al., 2021). Additionally, epigenetic modifications, including DNA methylation and histone acetylation, may further influence gene expression under varying soil and climatic conditions (Sun et al., 2021). While qRT-PCR validation was not performed in this study, the robustness of our RNA-seq results is supported by stringent statistical criteria (FDR-adjusted *P* < 0.05). Future studies will focus on experimental validation of key DEGs and explore the roles of TFs and epigenetic regulation in bamboo shoot nutrition.

This study represents a significant advancement in understanding the genetic mechanisms underlying bamboo shoot quality. By integrating transcriptomics with environmental data, we identified specific genes and pathways that regulate nutrient accumulation, filling a critical gap in previous research. While studies on Moso bamboo have explored light-induced lignin-related gene expression (Li et al., 2023b), this is the first comprehensive transcriptome-wide analysis of Lei bamboo nutrition. Our findings provide a foundation for precision cultivation strategies, enabling the optimization of bamboo shoot quality through targeted genetic and environmental interventions.

### 4.4 Building a model linking environmental factors, transcriptome, and nutritional traits

To elucidate the interactions between environmental factors, transcriptome changes, and nutritional traits, we developed a model using principal component analysis (PCA) and stepwise regression. The PCA model explained 69.05% of the total variance, with PC1 (44.47%) driven by soil organic matter, total phosphorus, and total nitrogen, and PC2 (24.58%) influenced by annual precipitation and longitude. These results underscore the dominant roles of soil fertility and climatic factors in shaping bamboo shoot nutrition.

The stepwise regression model exhibited high predictive accuracy for key nutritional traits, with *R²* values ranging from 0.72 to 0.85 for soluble sugars, cellulose, and free amino acids. Soil organic matter and porosity were the primary drivers of soluble sugar and starch accumulation, linked to the upregulation of starch metabolism genes (*e.g.*, *BGLU*, *SPS*, *SBE*, *SUS*). Conversely, higher porosity and lower organic matter in regions like Xixiang corresponded to increased cellulose content, associated with the upregulation of cellulose synthesis genes. Free amino acid levels showed a strong correlation with longitude and annual precipitation, with higher values in regions experiencing greater rainfall.

Validation using an independent dataset confirmed the model’s robustness, with *R²* values between 0.68 and 0.79. The model demonstrates that environmental variations, particularly in soil fertility and geographical factors, modulate gene expression in key metabolic pathways, thereby shaping the nutritional profile of bamboo shoots. This integrative approach provides a foundation for precision cultivation strategies aimed at optimizing bamboo shoot quality across different regions.

However, the model has limitations. It is region-specific and may not fully capture environmental variability in other cultivation areas. Additionally, genetic variation among bamboo species could limit its applicability. For example, *Phyllostachys edulis* (Moso bamboo) may exhibit different metabolic pathways compared to *Phyllostachys violascens* (Lei bamboo). Future studies should expand the model to include multiple bamboo species and a wider range of environmental conditions. Integrating machine learning algorithms and non-linear modeling approaches could further enhance the model’s ability to capture complex interactions.

Despite these limitations, the current model provides valuable insights for optimizing bamboo shoot quality in regions with similar environmental and genetic backgrounds, supporting precision cultivation practices and sustainable bamboo production. By identifying key environmental drivers and transcriptomic changes, this study offers a scientific foundation for tailoring cultivation strategies to enhance the nutritional quality of bamboo shoots.

## 5 Conclusion

This study highlights the significant role of environmental factors in shaping the nutritional composition of bamboo shoots. Climate, soil properties, and geographical conditions were identified as key drivers of regional variation in bamboo shoot quality. Soil organic matter, total nitrogen, potassium, and available phosphorus were found to be crucial in nutrient accumulation, with notable differences observed in levels of soluble sugars, cellulose, lignin, and secondary metabolites such as phenolics and flavonoids. Longitude emerged as a key geographical factor influencing amino acid and phenolic content, with areas of higher precipitation promoting amino acid synthesis and accumulation. Transcriptome analysis revealed that environmental factors regulate the expression of genes involved in starch metabolism, phenylpropanoid biosynthesis, and flavonoid biosynthesis, providing molecular insights into how bamboo shoots adapt to varying conditions. The study proposes a model linking environmental factors to gene expression and nutritional traits, offering a framework for optimizing bamboo shoot quality through precision cultivation and tailored regional management. While the results provide a comprehensive understanding of how environmental factors affect bamboo shoot nutrition, further studies are needed to explore the genetic mechanisms more thoroughly. Future research should focus on identifying genetic markers for key nutritional traits and uncovering the regulatory networks between genes and environmental factors. Additionally, examining the long-term effects of climate change and fluctuating environmental conditions on bamboo shoot nutrition will help enhance our understanding of bamboo’s adaptability and resilience.

In conclusion, these findings offer essential insights for the sustainable cultivation of bamboo shoots, underscoring the importance of region-specific environmental management to enhance nutritional value and meet consumer preferences for healthier bamboo-based products. The results also provide strategies to improve the commercial value of bamboo shoots by optimizing cultivation practices in response to environmental variability.

## Data Availability

The datasets presented in this study can be found in online repositories. The names of the repository/repositories and accession number(s) can be found below: https://www.ncbi.nlm.nih.gov/, PRJNA1214042.
