# Supplementary material for: Environmental drivers and transcriptomic variations shaping Lei bamboo shoots across cultivation regions
Source: Front Plant Sci. 2025 Apr 1;16:1565665. doi: 10.3389/fpls.2025.1565665 (PMC11997477; doi:10.3389/fpls.2025.1565665)
Supplement: Supplementary file 1 [file DataSheet1.docx]

Supplementary Material

# Supplementary Data

Supplementary Material should be uploaded separately on submission. Please include any supplementary data, figures and/or tables.

Supplementary material is not typeset so please ensure that all information is clearly presented, the appropriate caption is included in the file and not in the manuscript, and that the style conforms to the rest of the article.

# Supplementary Figures and Tables

## Supplementary Figures


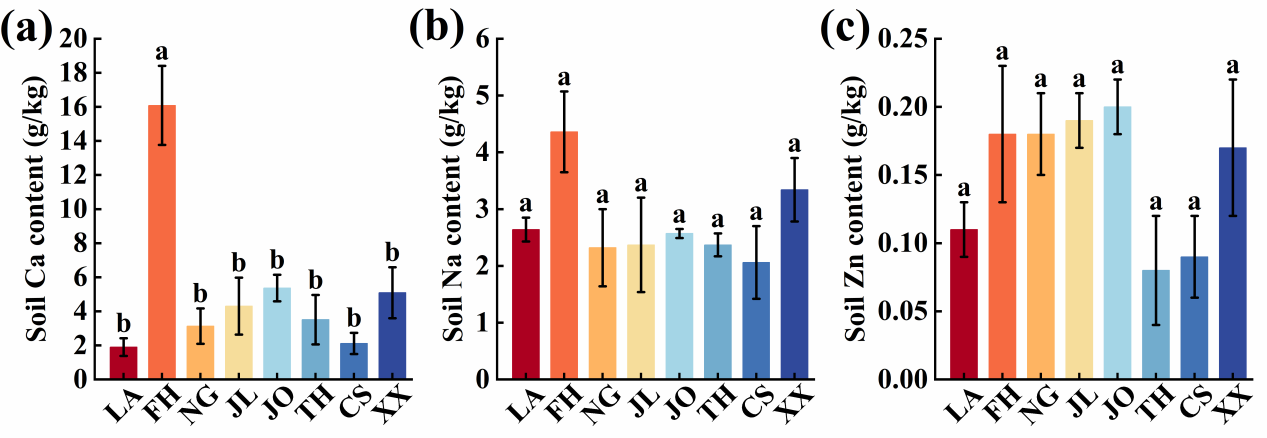


**Figure S1 Soil physicochemical properties of Phyllostachys praecox (LA) transplanted to different cultivation regions. LA represents the Taihuyuan site in Lin'an, Hangzhou, Zhejiang Province; NG represents the Ningguo site in Xuancheng, Anhui Province; FH represents the Fenghua site in Ningbo, Zhejiang Province; JL represents the Jiaoling site in Meizhou, Guangdong Province; JO represents the Jian'ou site in Nanping, Fujian Province; TH represents the Taihe site in Ji'an, Jiangxi Province; CS represents the Chishui site in Zunyi, Guizhou Province; XX represents the Xixiang site in Hanzhong, Shaanxi Province.**


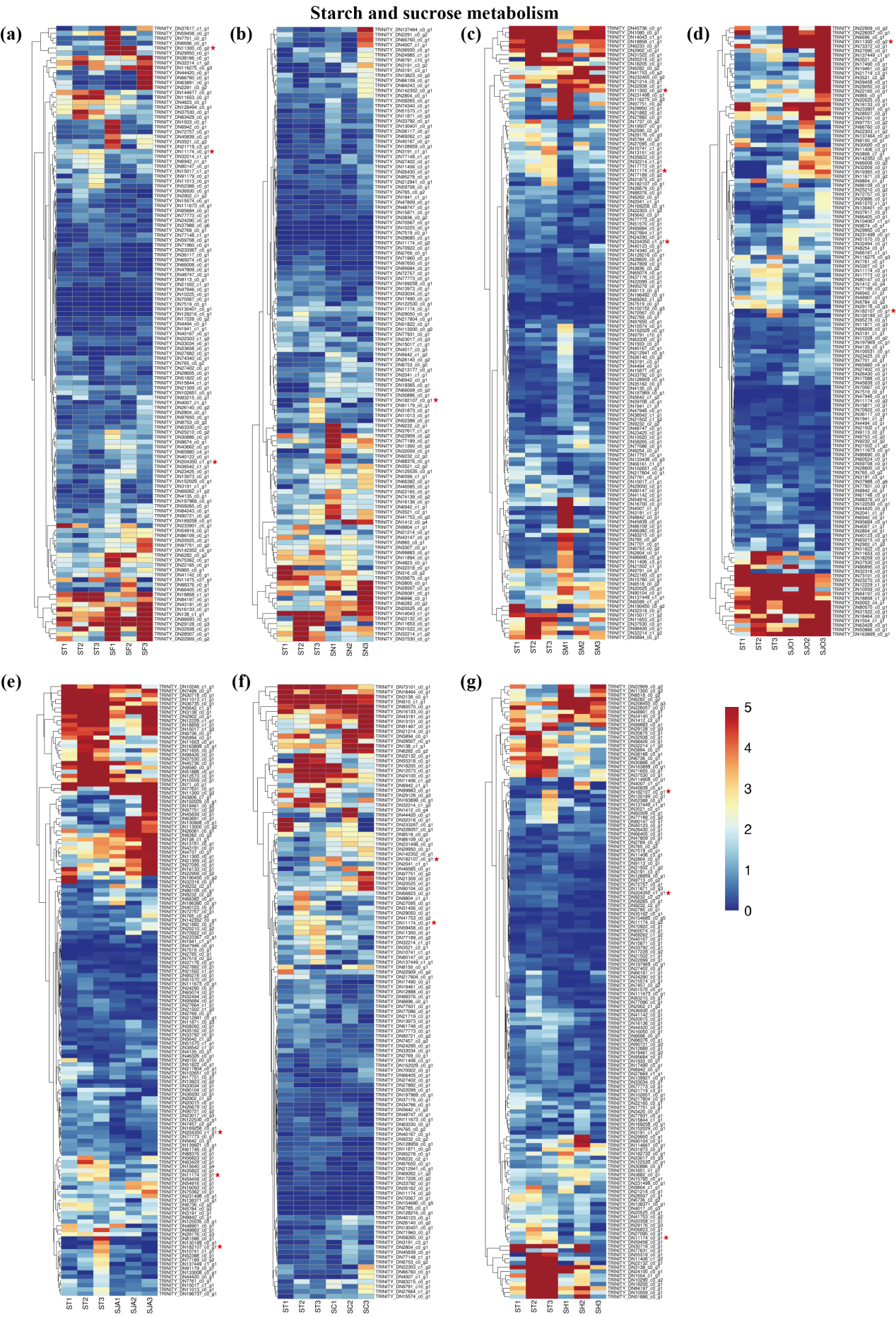


**Figure S2 The color of the cells in the heat map indicates the level of starch and sucrose metabolism gene expression. ST represents the transcriptome of bamboo shoots from Lin'an; SF represents those from Fenghua; SN from Ningguo; SM from Jiaoling; SJO from Jian'ou; SJA from Taihe; SC from Chishui; and SH from Xixiang.**


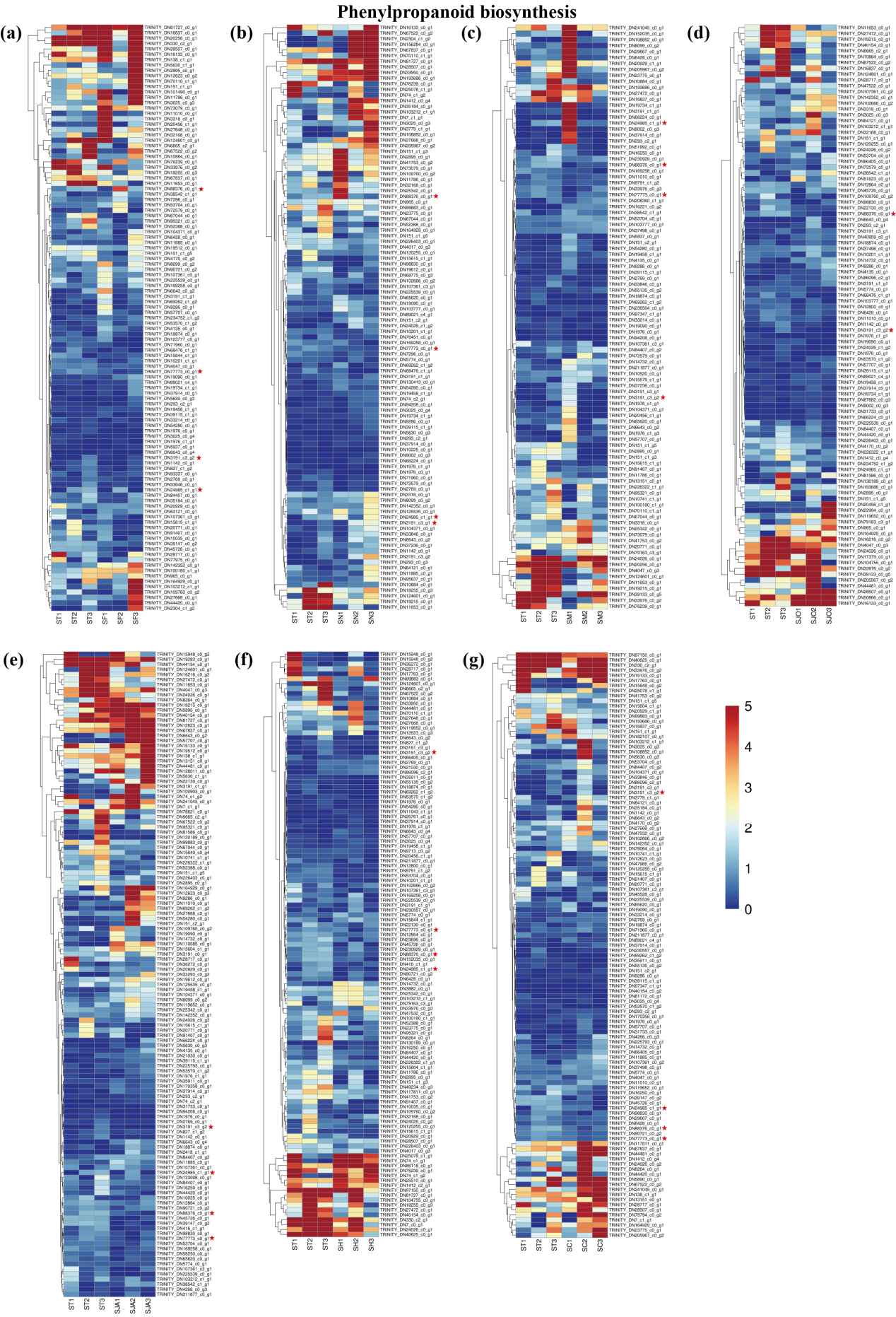


**Figure S3 The color of the cells in the heat map indicates the level of phenylpropanoid biosynthesis gene expression. ST represents the transcriptome of bamboo shoots from Lin'an; SF represents those from Fenghua; SN from Ningguo; SM from Jiaoling; SJO from Jian'ou; SJA from Taihe; SC from Chishui; and SH from Xixiang.**


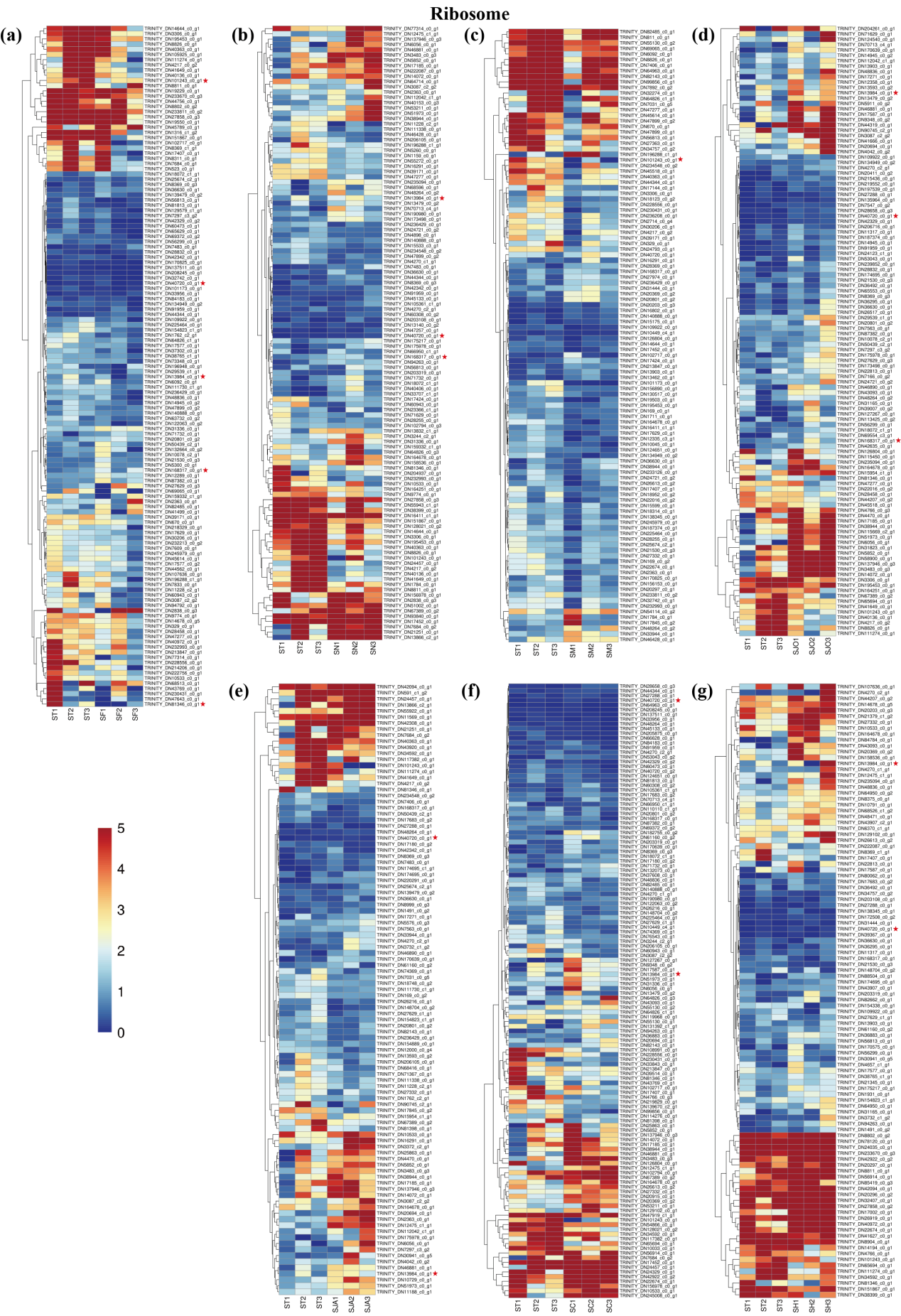


**Figure S4 The color of the cells in the heat map indicates the level of ribosome gene expression. ST represents the transcriptome of bamboo shoots from Lin'an; SF represents those from Fenghua; SN from Ningguo; SM from Jiaoling; SJO from Jian'ou; SJA from Taihe; SC from Chishui; and SH from Xixiang.**


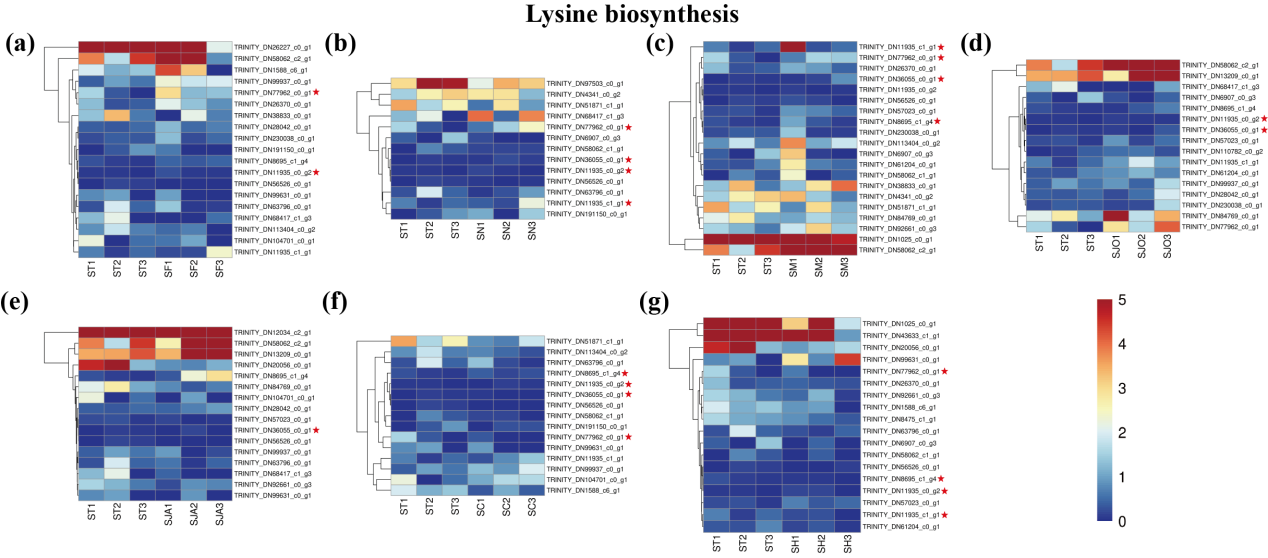


**Figure S5 The color of the cells in the heat map indicates the level of lysine biosynthesis gene expression. ST represents the transcriptome of bamboo shoots from Lin'an; SF represents those from Fenghua; SN from Ningguo; SM from Jiaoling; SJO from Jian'ou; SJA from Taihe; SC from Chishui; and SH from Xixiang.**


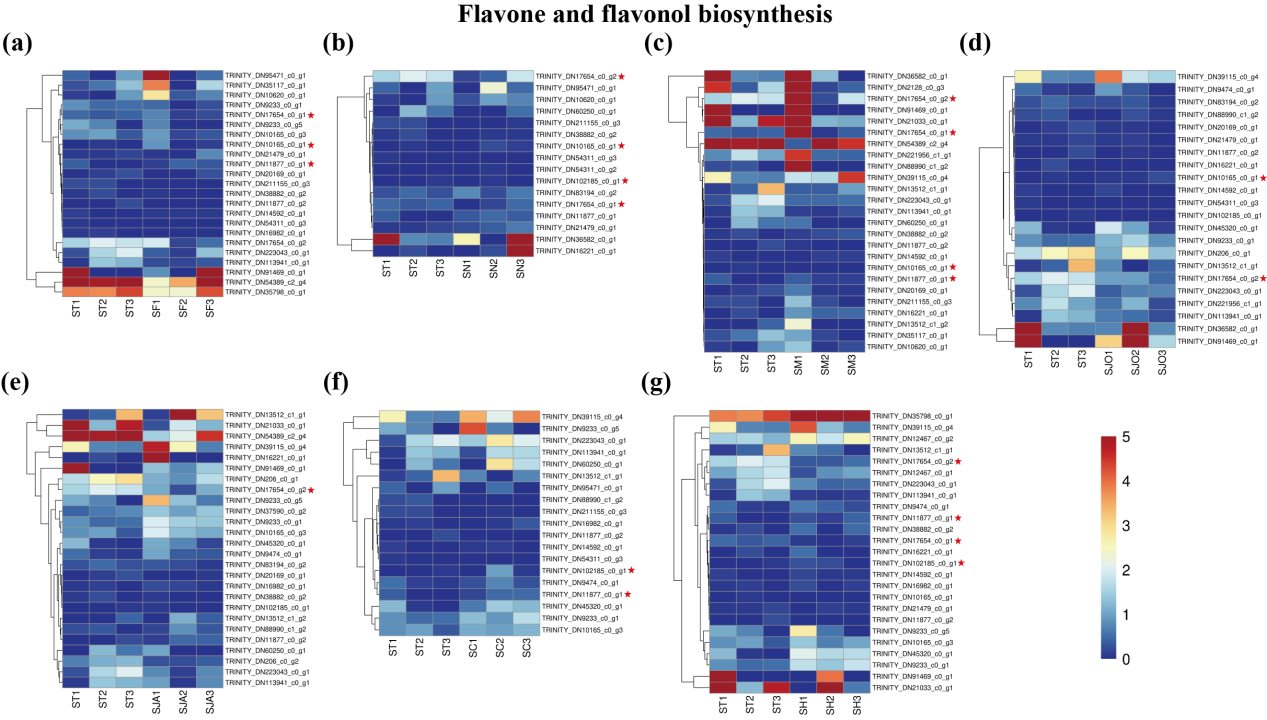


**Figure S6 The color of the cells in the heat map indicates the level of flavone and flavonol biosynthesis gene expression. ST represents the transcriptome of bamboo shoots from Lin'an; SF represents those from Fenghua; SN from Ningguo; SM from Jiaoling; SJO from Jian'ou; SJA from Taihe; SC from Chishui; and SH from Xixiang.**

## Supplementary Tables

**Table S1 Soil Types in the Study Areas****. LA represents the Taihuyuan site in Lin'an City, Hangzhou, Zhejiang Province. NG represents the Ningguo site in Xuancheng City, Anhui Province. FH represents the Fenghua site in Ningbo City, Zhejiang Province. JL represents the Jiaoling site in Meizhou City, Guangdong Province. JO represents the Jianou site in Nanping City, Fujian Province. TH represents the Taihe site in Ji'an City, Jiangxi Province. CS represents the Chishui site in Zunyi City, Guizhou Province. XX represents the Xixiang site in Hanzhong City, Shaanxi Province.**

| Sample | Clay %  (<0.002mm) | Silt %  （0.002~0.02mm**）** | Sand %  （0.02~0.2mm**）** | Coarse sand %  （0.2-2mm**）** | Soil type |
| --- | --- | --- | --- | --- | --- |
| LA | 39.8 | 41.47 | 18.58 | 0.15 | Loamy clay |
| NG | 32.91 | 43.89 | 21.93 | 1.27 | Loamy clay |
| FH | 42.06 | 28.89 | 28.31 | 0.76 | Loamy clay |
| JL | 37.36 | 38.31 | 23.43 | 0.9 | Loamy clay |
| JO | 30.26 | 37.99 | 31.71 | 0.04 | Loamy clay |
| TH | 36.85 | 36.85 | 26.12 | 0.19 | Loamy clay |
| CS | 42.65 | 29.99 | 27.18 | 0.18 | Loamy clay |
| XX | 27.1 | 40.49 | 30.34 | 2.07 | Loamy clay |
